# The Role of lncRNA PCAT6 in Cancers

**DOI:** 10.3389/fonc.2021.701495

**Published:** 2021-07-13

**Authors:** Siying Wang, Zhenyao Chen, Jingyao Gu, Xin Chen, Zhaoxia Wang

**Affiliations:** Cancer Medical Center, The Second Affiliated Hospital of Nanjing Medical University, Nanjing, China

**Keywords:** PCAT6, lncRNA, cancer, gene regulation, pathway

## Abstract

Long non-coding RNA (lncRNA) PCAT6 is a member of the Prostate Cancer Associated Transcripts family of molecules. In this review, we focus on the latest studies involving PCAT6 in the diagnosis, treatment, and prognosis of malignant tumors of the digestive, respiratory, urinary, reproductive, motion, and nervous systems. PCAT6 was found to be highly expressed in gastric cancer, colon cancer, hepatocellular carcinoma, lung cancer, bladder cancer, ovarian cancer, breast cancer, cervical cancer, osteosarcoma, glioblastoma, and other tumors. PCAT6 can promote the development and progression of different types of malignant tumors through various mechanisms. Overall, these findings suggest that PCAT6 may play an increasingly vital role in the clinical assessment of these malignant tumors. It can function as an oncogene and may be used as a potential new prognostic biomarker of these tumors.

## Introduction

According to the human genome project, protein-coding genes account for less than 2% of the entire genome, while the remaining DNA is non-coding. Long non-coding RNAs (lncRNAs) are RNA transcripts longer than 200 nucleotides. More than 10,000 lncRNAs have been discovered in humans and mice in recent years. Various studies have shown that lncRNAs play central roles in tumor biology *via* different molecular mechanisms, including tumor invasion, metastasis, and even multi‐drug resistance (1–3). LncRNAs are reported to have a variety of regulatory forms during cancer progression. For example, lncRNAs can work as sponges by competitively binding to microRNAs (miRNAs) or protein complexes. Our group also focused on the regulatory mechanisms of lncRNAs. As shown in our previous study, the LINC01234-miR-340-5p/miR-27b-3p-VAV3 axis plays important roles in the progression of non-small cell lung cancer (NSCLC) (4). Moreover, LINC01234 promotes cells proliferation *in vitro* and tumor growth *in vivo* by acting as a competing endogenous RNA (ceRNA) for miR-204-5p and regulating core-binding factor β (CBFB) expression in gastric cancer (GC) (5). Furthermore, LINC00152 promotes lung adenocarcinoma (LUAD) cell proliferation by interacting with the enhancer of zeste homolog 2 (EZH2) and repressing interleukin (IL)-24 expression (6). LncRNAs may also regulate pathophysiological processes by transcriptional activation or interference. Additionally, lncRNAs are associated with a wide spectrum of biological processes including imprinting, pluripotency, cell cycle regulation, and retrotransposon silencing (7). LncRNAs are also associated with many diseases. Disease-related lncRNAs will gain greater relevance as potential biomarkers in cancers and for personalized medicine, especially for gene therapy (8).

**Figure 1:**
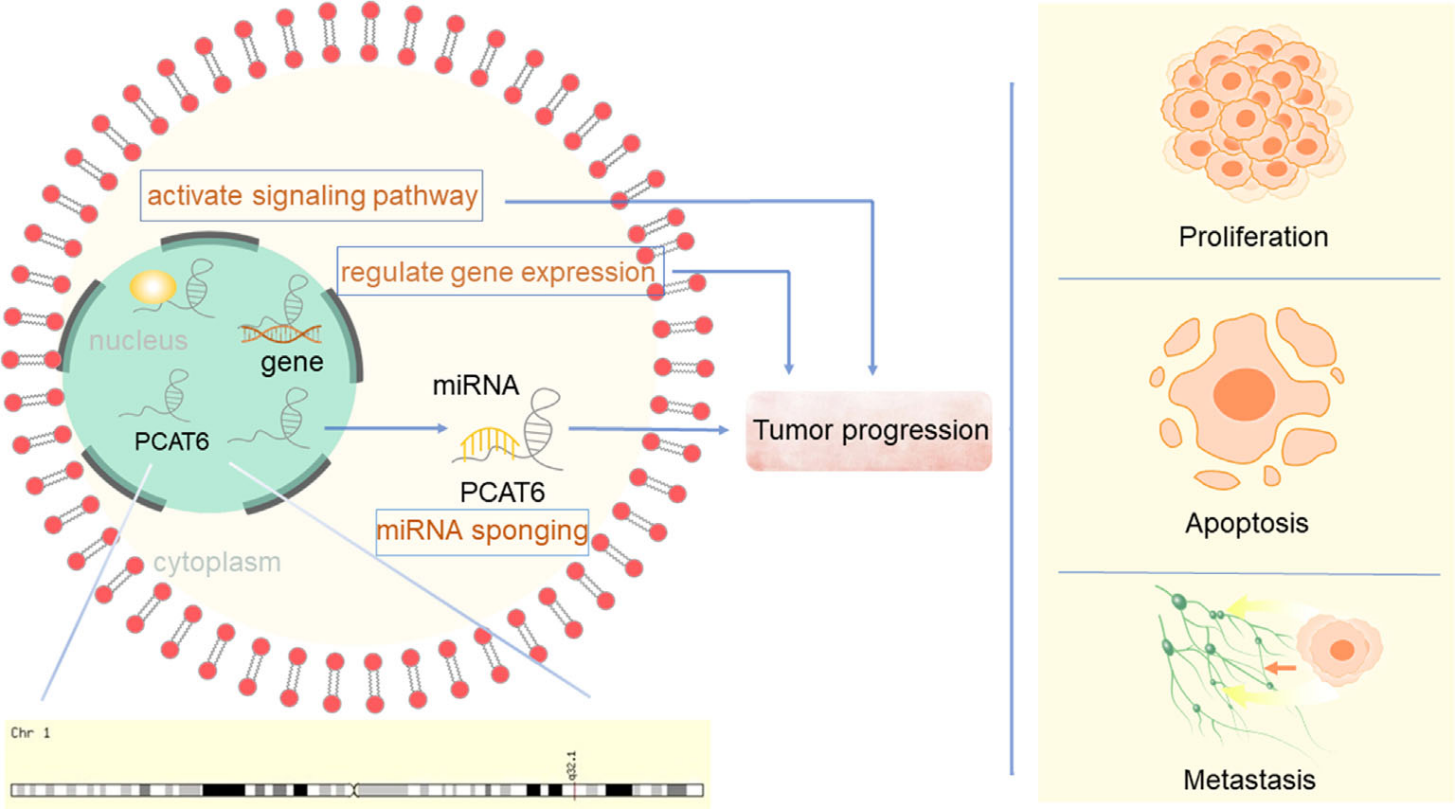
LncRNA PCAT6 promote cancer progression through different mechanism.

Prostate Cancer Associated Transcript 6 (PCAT6), also known as PCAN-R1, ncRNA-a2, and KDM5B-AS1, is a newly discovered carcinogenic lncRNA. The PCATs form a series of 121 lncRNAs that were first detected in prostate cancer using computational bioinformatics means to delineate the annotated and unannotated transcripts in this disease (9). PCAT6 was identified as a 764 bp-long, intergenic lncRNA located on chromosome 1q32.1, flanking the histone demethylase JARID1B/KDAM 5B. It was first found to induce keratinocyte proliferation and colony formation of prostate tumor cells (10). PCAT6 was determined to be mainly localized to the nucleus (11). Since its identification, PCAT6 has been suggested to be oncogenic and promote tumor progression *via* different mechanisms in various carcinomas (**Figure 1**), including lung cancer, GC, colorectal cancer (CRC), hepatocellular carcinoma (HCC), bladder cancer (BC), breast cancer, cervical cancer (CC), and osteosarcoma. Although only a small number of functions of PCAT6 have been reported, PCAT6 has become known as an essential regulator in biology and is involved in controlling various physiological processes. To date, the precise molecular mechanism of PCAT6 in cancers remains unclear. Here, we review the regulatory mechanisms of PCAT6 in different cancers, and in-depth study is expected to provide new therapeutic targets for these diseases.

## The Role of PCAT6 in Cancer Cells

### PCAT6 in Carcinomas

#### Lung Cancer

Lung cancer is one of the most common malignancies worldwide. About 25% of cancer deaths are related to lung cancer (12). Even as we learn more about the development, risk, and treatment options of lung cancer, it remains the main cause of cancer-related deaths. Smoking is still the major risk factor for lung cancer development (13). Although many treatments are effective in improving the overall survival (OS) of these patients, further understanding of the molecular mechanisms and identification of new therapeutic targets are still urgently needed.

Data from The Cancer Genome Atlas (TCGA) and Gene Expression Omnibus (GEO) indicated that PCAT6 is upregulated in human NSCLC tissues (11) compared with normal human bronchial epithelial (NHBE) cells, especially in CL1-5 and H446 cells (14). Twelve targets were successfully validated by qRT-PCR, and PCAT6 was found to be upregulated in squamous cell carcinomas than in adenocarcinomas (15). Analysis of five GEO datasets suggested that the sensitivity and specificity of PCAT6 were significantly higher than those of carcinoembryonic antigen (CEA), which had a sensitivity of 51.1% and a specificity of 84.8% in NSCLC. Plasma from 73 LUAD patients, 51 lung squamous cell carcinoma (LUSC) patients, and 39 normal healthy donors revealed that plasma expression of PCAT6 was significantly increased in LUAD and LUSC patients. Furthermore, plasma PCAT6 expression was positively correlated with the metastasis status and TNM stage of LUAD and LUSC tumors (16). Shi et al. (11) suggested that PCAT6 overexpression could promote tumor cell growth in NSCLC, while knockdown of PCAT6 could mitigate NSCLC cell growth *via* induction of G1-phase cell cycle arrest and apoptosis. PCAT6 could also promote NSCLC cell migration and invasion. Therefore, PCAT6 could possibly be an important factor that contributes to NSCLC progression. Functionally, increased PCAT6 levels were positively associated with tumor size, TNM stage, lymph node metastasis, and poor OS (14).

The carcinogenic activity of PCAT6 was strongly correlated with the NSCLC pathways (17). First, Shi et al. (11) revealed a potential molecular mechanism that PCAT6 silences LATS2 transcription by binding to H3K27 methyltransferase EZH2. Second, Wan et al. (14) suggested that PCAT6 can regulate the expression of pivotal cancer-related proteins c-Myc and p53 in lung cancer cells. Knockdown of PCAT6 significantly decreased c-Myc expression and enhanced p53 expression. Although RNA immunoprecipitation experiments revealed that PCAT6 did not directly bind to these proteins, PCAT6 knockdown noticeably affected their expression levels. Additionally, Cui et al. (18) indicated that miR-330-5p is a target of PCAT6. PCAT6 could promote NSCLC cell progression by competitively binding miR-330-5p. Knockdown of PCAT6 could regulate miR-330-5p, resulting in inhibited proliferation, migration, and invasion of NSCLC cell lines.

#### Gastric Cancer

GC is a malignant disease with high incidence around the world (19). It is also the most common gastrointestinal cancer in East Asia (20), with a 5-year OS rate of less than 10% (21). Therefore, a stronger understanding of the pathogenesis of GC is very important.

qRT-PCR analysis revealed that the expression levels of PCAT6 were significantly upregulated in 20 GC tissues compared with those in paired adjacent normal tissues. Silencing of PCAT6 significantly restrained the epithelial-mesenchymal transition (EMT) and proliferation of GC cells (22). PCAT6 expression was also negatively correlated with tumor size, TNM stage, metastasis status, and GC prognosis (23). In addition, PCAT6 could bind some miRNAs and exert its role as a ceRNA. Several pathways are involved in the carcinogenic effects of PCAT6. For example, silencing of PCAT6 could reduce the protein levels of members of the RB/E2F and Wnt/β-catenin signaling pathways by upregulating miR-15a (22). PCAT6 can also endogenously compete with miR-30 by targeting MKRN3. Both PCAT6 overexpression and miR-30 knockdown could promote MKRN3 expression (23).

The authors of these PCAT6 studies propose possible approaches for the clinical treatment of GC. By regulating the expression of PCAT6 in GC, cancer-associated protein levels will be affected *via* signaling pathways. Suppressing PCAT6 expression may also inhibit the progression of GC.

#### Colorectal Cancer

The development of CRC is associated with many factors, such as lifestyle, an unhealthy diet, metabolic disorders, heredity, and genetic factors (24). In developed countries, CRC mortality rates have been decreasing; however, in developing countries, these rates are trending upwards (25). Although the prognosis for early stage CRC is excellent, the 5-year survival rate in metastatic disease remains low (26).

Analysis of 58 cases of colon cancer and 439 RNA-seq transcriptome profiles from the TCGA-colon adenocarcinoma (COAD) dataset showed that PCAT6 was significantly upregulated. High levels of PCAT6 were remarkably associated with the tumor subtype, N classification, metastasis status, clinical stage, vital status, and a worse overall patient survival (27). Moreover, another study indicated that PCAT6 expression was much higher in samples of advanced TNM stages (III + IV) than that in early stages (I + II) (28). Next, PCAT6 could promote CRC cell growth and inhibit apoptosis *in vitro*. Furthermore, a xenograft mouse model and lentiviral infection of SW620 cells (SW620-LV-PCAT6) revealed that PCAT6 contributes to the tumorigenesis of CRC cells *in vivo*. Importantly, gene set enrichment analysis (GSEA) and TCGA data profiles, combined with chromatin immunoprecipitation (ChIP) assays, indicated that PCAT6 interacts with EZH2 in a complex which acts as a key activator of apoptosis repressor with caspase (ARC) expression and occupancy. The process can inhibit CRC cell apoptosis and contribute to disease progression (27). Luciferase reporter gene assays provided validation that PCAT6 can also interact with miR‐204 through direct binding. Immunoblotting and chemoresistance mechanistic studies have suggested that the elevated PCAT6 levels inhibit miR‐204 expression in CRC, promoting HMGA2/PI3K signaling pathway activity and enhancing the chemoresistance of CRC cells to 5‐fluorouracil (FU) (28).

#### Hepatocellular Carcinoma

HCC is one of the most prevalent and deadly cancers in the world. Chronic viral hepatitis and alcoholic/nonalcoholic steatohepatitis are its most well-defined risk factors (29). Many of the patients were at advanced stage of HCC, for which highly effective treatments are limited (30). Bioinformatics analysis confirmed that a total of 389 PCAT6-related genes were found in HCC tissues and cell lines (221 of 389 genes were differentially expressed), which were highly enriched in various key pathways (31).

Publicly available RNA-seq data of 374 HCC patients and 50 adjacent tissues from the TCGA-liver hepatocellular carcinoma (LIHC) dataset, combined with qRT-PCR data from 29 pairs of HCC tissues, revealed that PCAT6 expression was markedly upregulated in HCC tissues compared with adjacent tissues. Additionally, PCAT6 expression was higher in HepG2 and PLC/PRF/5 cells relative to other HCC cell lines, suggesting that PCAT6 possibly exerts an oncogenic effect in HCC development. Next, overexpression of PCAT6 can promote the proliferation of HCC cells, as well as inhibit cell cycle arrest and cell apoptosis. PCAT6 expression was also significantly correlated with poor differentiation and advanced TNM stage. Kaplan-Meier analysis indicated that patients with lower PCAT6 expression levels had significantly longer OS (P < 0.001) and disease-free survival (DFS) (P < 0.05), suggesting that PCAT6 overexpression is correlated with poor prognosis of HCC patients (31). Furthermore, HCC patients with higher expression levels of both LINC01138 and PCAT6 had poorer progression-free survival (PFS), and upregulated PCAT6 was correlated with shorter survival time (32).

Hanahan et al. (33) found that gene amplification of PCAT6 could induce the observed increase of PCAT6 expression in LIHC. The copy number amplification of oncogenes is widely recognized as a driver of cancer. Chen et al. (34) identified a key pathway involved – the activation of PCAT6 occurred together with numerous genes related to RNA processing and mitotic cell cycle. The authors also found that PCAT6 showed a remarkable negative association with CD34, which is a marker of stemness. The abovementioned conclusions suggest that PCAT6 has a possible oncogenic role in HCC, and is a candidate driver of HCC development.

#### Bladder Cancer

BC is considered to be the most common genitourinary malignancy worldwide (35, 36). Despite great progress being made in the treatment of BC in recent years, the incomplete understanding of BC mechanisms causes patients with advanced disease to still have poor prognoses (37).

Xia et al. (38) used qRT-PCR to analyze PCAT6 expression in a total of 21 paired BC tissues and adjacent normal tissues. PCAT6 expression was upregulated, while miR-513a was downregulated, in BC cells. PCAT6 knockdown inhibited the viability of BC cells and significantly restrained their migration and invasion rates. Additionally, PCAT6 inhibited miR-513a expression through direct interaction and promoted BC progression by acting as a miR-513a sponge. Moreover, Kaplan-Meier analysis revealed that BC patients with low PCAT6 expression showed longer OS times compared with those patients with high PCAT6 expression. These results indicate that upregulated PCAT6 could predict poor prognosis in BC patients.

Similarly, Zhang et al. (39) analyzed TCGA-Urothelial Bladder Carcinoma (BLCA) data and found that PCAT6 expression was upregulated in BC tumor samples compared with non-tumor samples. Serum PCAT6 levels were also higher in BC patients than in healthy controls. Furthermore, OS and PFS were shorter in BC patients with high PCAT6 expression. Correlation analyses showed that PCAT6 expression is related to tumor size, differentiation, TNM stage, lymph node metastasis status, and distant metastasis status, but not gender or age.

#### Breast Cancer

Breast cancer has become the leading cause of cancer-related mortality among women in the world (40). During the past several decades, its diagnosis and treatment have developed rapidly, so its incidence and mortality rates are expected to increase noticeably in the coming years (41).

Triple-negative breast cancer (TNBC) is characterized by the absence of the PR, ER, and HER2 receptors. A subcellular fractionation and FISH assay indicated that PCAT6 was distributed in both the cytoplasm and nucleus of TNBC cells. PCAT6 levels were significantly increased in 86 TNBC tissues compared with those in paired adjacent noncancerous nontumor tissues, and metastatic tissues contained higher PCAT6 expression than non-metastatic tissues. Moreover, additional assays demonstrated that depletion of PCAT6 could attenuate cell proliferation. Transwell assays indicated that silenced PCAT6 remarkably reduced cell invasion, while wound-healing assays revealed that it hampered cell migration. Western blot analysis suggested that PCAT6 knockdown resulted in a marked increase in E-cadherin protein levels, but decreased Slug, N-cadherin, and Twist protein levels. These data suggest that PCAT6 can facilitate cell proliferation, invasion, migration, and the EMT process. Next, M2 macrophage secreted vascular endothelial growth factor (VEGF) to upregulate PCAT6 and promote angiogenesis in TNBC. Through bioinformatics analysis and mechanistic assays, PCAT6 was found to act as a sponge for miR-4723-5p to upregulate VEGFR2, stabilize VEGFR2 by recruiting USP15, and participate in the VEGFR/AKT/mTOR signaling pathway to accelerate angiogenesis. Overall, M2 macrophages could induce PCAT6 upregulation which promoting TNBC tumorigenesis through modulation of VEGFR2 expression *via* ceRNA and deubiquitination mechanisms (42).

Additional studies using CCK-8 assays and qRT-PCR analyses have suggested that knockdown of PCAT6 can promote TNBC cell radiosensitivity, as well as elevate sensitivity to ionizing radiation, by promoting cell apoptosis and inhibiting cell survival. MiR-185-5p was confirmed to be a downstream target of PCAT6, indicating that it acts as a molecular sponge for this miRNA. This negative modulation of miR-185-5p expression ultimately affects TPD52 expression. The high expression of PCAT6 was also correlated with aggressive tumor phenotype, affecting clinical stage and lymph node metastasis (43).

#### Cervical Cancer

CC is one of the most common cancers in women. Human papillomavirus (HPV) infection remains the leading cause of CC (44). Despite recent progress in surgical, chemotherapeutic, and radiotherapy methods, CC patient prognosis remains poor. Therefore, further study is needed to elucidate more detailed mechanisms.

qRT-PCR was used to detect PCAT6 expression in CC, and the results showed that it was significantly elevated in CC tissues compared with normal cervical tissues and closely correlated with CC progression. Patients with high PCAT6 expression had shorter OS and DFS. Silencing PCAT6 impaired the migration and invasion of CC cells. High expression of PCAT6 acts as an independent indicator of unfavorable prognosis and increased PCAT6 was markedly correlated with advanced FIGO stage, lymph node metastasis status, and depth of cervical invasion (45).

Mechanistically, TOP/FOP flash reporter assays and qRT-PCR analyses suggested that PCAT6 positively regulates the Wnt/β-catenin signaling pathway in CC cell lines by promoting the expression of c-myc, cyclin D1, and β-catenin (45). Next, Starbase bioinformatic software predicted that miR-543 is a target of PCAT6. PCAT6 can negatively regulate miR-543 expression as a sponge in CC cells. Upregulated PCAT6 promotes the proliferation, metastasis, and chemoresistance of CC cells. Moreover, ZEB1 was predicted to be a target of miR-543 based on Starbase information. There was a positive relationship between the enrichment of PCAT6 and ZEB1 mRNA expression levels in CC tissues. PCAT6 could promote proliferation and metastasis, as well as inhibit apoptosis, in CC cells by modulating the PCAT6/miR-543/ZEB1 axis. MTT assays showed that PCAT6 can also facilitate the chemoresistance of CC cells to cisplatin *via* this axis (46).

#### Other Cancers

In addition to the common carcinomas described above, PCAT6 is relevant in other carcinomas. In prostate cancer, PCAT6 was indicated as the most upregulated lncRNA in cancer tissues and was also correlated with metastasis status. Furthermore, PCAT6 could enhance prostate cancer cell proliferation and colony formation in an androgen-independent manner (10). In cholangiocarcinoma, PCAT6 directly targeted and reduced miR-330-5p levels, resulting in induced cell proliferation and invasion (47). In pancreatic ductal adenocarcinoma (PDAC), PCAT6 upregulated oncogene CBX2 expression by sponging miR-185-5p. Thus, the PCAT6/miR-185-5p/CBX2 pathway possibly has crucial functions in tumorigenesis and progression of PDAC (48). In ovarian cancer, PCAT6 potentially promotes cell proliferation, migration, and invasion by inhibiting PTEN (49). In glioblastoma multiforme (GBM), PCAT6 could upregulate IGF2BP1 expression by acting as a ceRNA against miR-513, causing a PCAT6/miR-513/IGF2BP1 positive feedback loop that facilitates GBM progression (50).

### PCAT6 in Sarcomas

#### Osteosarcoma

Osteosarcoma is the most common primary bone cancer in children and adolescents and the third most common in adults (51). Younger age is a frequent risk factor, as well as race and gender (52). LncRNAs also play an important regulatory role in the proliferation, apoptosis, and differentiation of osteosarcoma cells (53). It is pivotal for us to further understand the molecular mechanisms involved in osteosarcoma.

PCAT6 was upregulated in osteosarcoma tissues compared with adjacent normal tissues, as seen by qRT-PCR assays. High expression of PCAT6 was positively correlated with advanced stage and metastasis status of osteosarcoma. PCAT6 can promote osteosarcoma cell proliferation, migration, and invasion. Furthermore, survival analyses indicated that PCAT6 upregulation contributed to shorter OS and PFS, indicating a poor prognosis (54).

Several PCAT6-associated pathways have been reported in osteosarcoma. Zhu et al. (54) demonstrated that PCAT6 acts as a ceRNA by sponging miR-185-5p, which results in upregulated TGFBR1 and TGFBR2 expression leading to TGF-β pathway activation. Next, Sun et al. (55) indicated that ZEB1 is a target of miR-143-3p. Mechanistic studies revealed that PCAT6 can increase ZEB1 levels by sponging endogenous miR-143-3p, and the upregulation of ZEB1 can aggravate the malignant phenotype of osteosarcoma cells. Therefore, PCAT6 plays a vital tumor-promoting role, partially dependent on regulation of the miR-143-3p/ZEB1 axis (56). In addition, Wu et al. (57) showed that overexpression of PCAT6 can promote MDM2 expression, while the levels of P53 and P21 were decreased. MDM2 knockdown could inhibit the proliferation, migration, and invasion of osteosarcoma cells.

#### Gastrointestinal Stromal Tumor

Gastrointestinal stromal tumor (GIST) is a rare cancer of mesenchymal origin that arises in the gastrointestinal tract and is often seen in adults and the elderly (58). It also has high rates of metastasis and recurrence, and metastases usually occur in the abdominal cavity or liver (59).

Bai. et al. (60) found that PCAT6 was remarkably upregulated in GIST tissues and cells compared with matched normal control groups in 72 pairs of GIST tissue samples. The authors performed bioinformatics analysis and experimentally verified that PCAT6 can upregulate PRDX5 by sponging miR‐143‐3p and activating the Wnt/β‐catenin pathway. Further experiments, like colony formation, JC‐1, and sphere formation assays, revealed the importance of PCAT6 in promoting cell proliferation and stemness, as well as repressing cell apoptosis.

## Discussion

LncRNAs have been demonstrated to be key regulators of human gene expression (61). Experts have used various methods to verify that PCAT6 is highly expressed in a variety of carcinoma and sarcoma tissues and cells. These findings are consistent with the results of multiple tumor cell functional experiments, such as proliferation, migration, and invasion assays.

**Figure 2:**
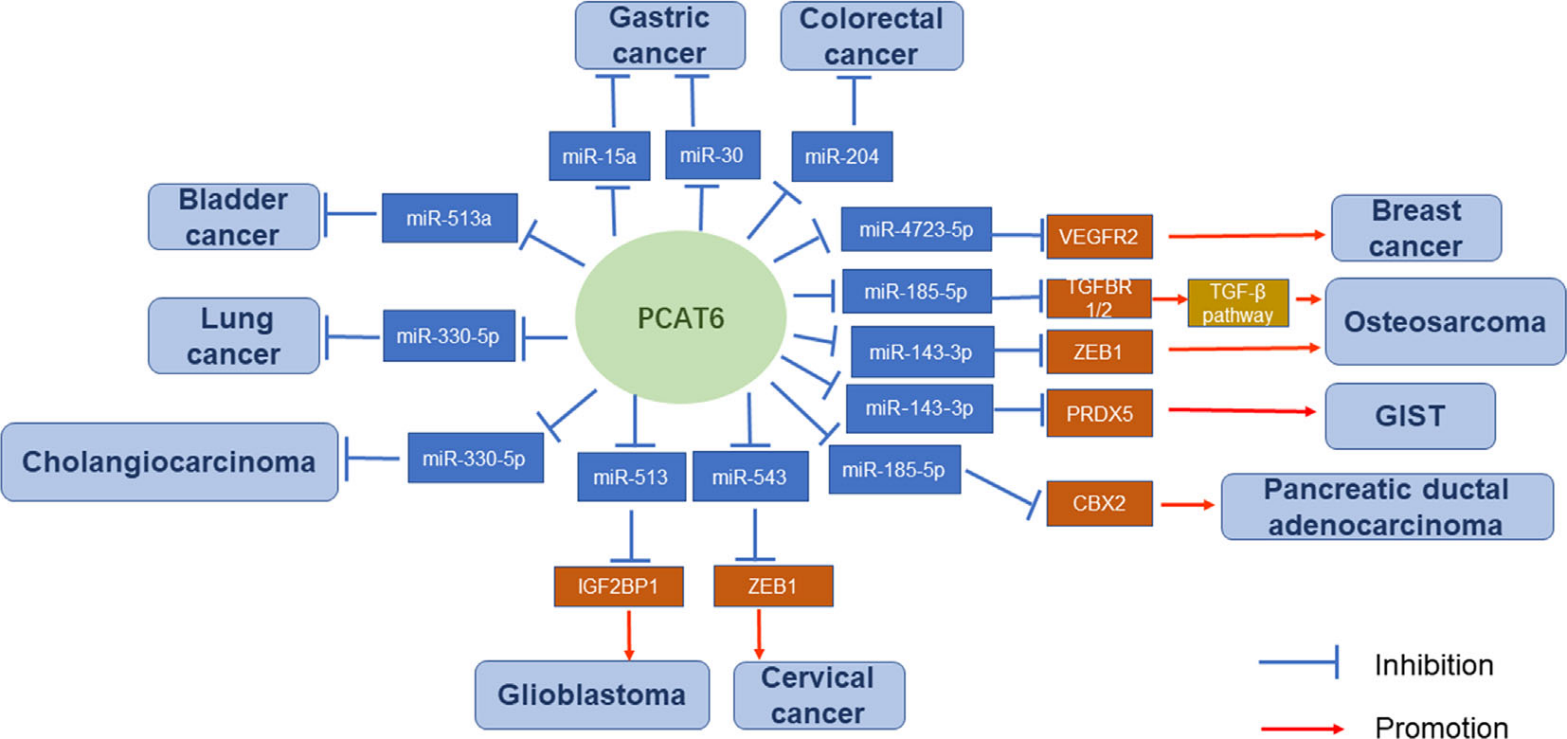
Overview of lncRNA PCAT6 binding to miRNAs.

**Figure 3:**
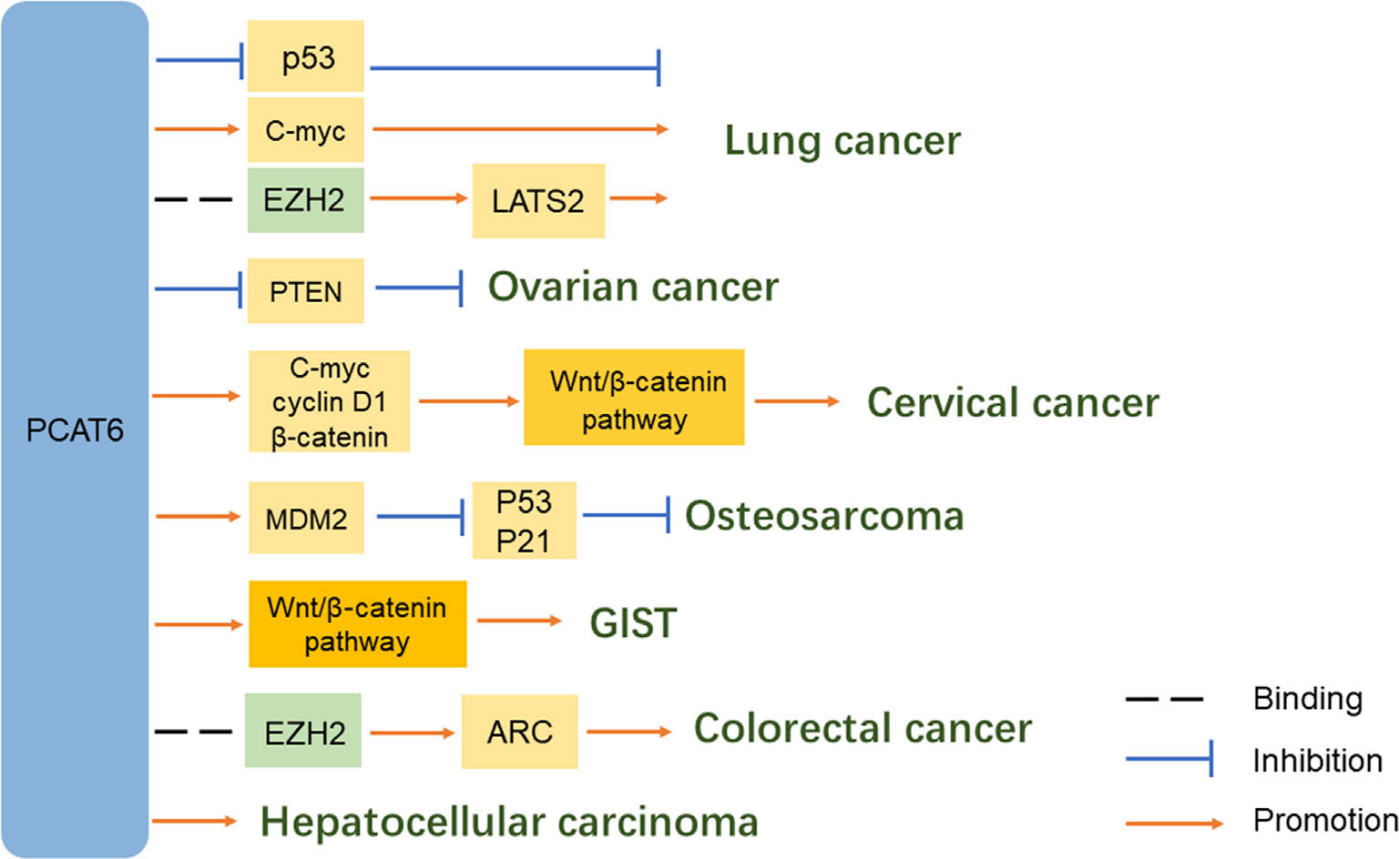
Role of lncRNA PCAT6 in carcinogenic signaling pathways.

Overall, the regulatory mechanisms of PCAT6 are very complex (**Figures 2** and **3**). PCAT6 targets the same miRNA in some tumors. For example, PCAT6 targets miR-185-5p in osteosarcoma and PDAC, miR-330-5p in lung cancer and cholangiocarcinoma, and miR-143-3p in GIST and osteosarcoma. In addition, PCAT6 mediates the same signaling pathways in some tumors, like how PCAT6 can mediate the Wnt/β-catenin pathway in GIST and osteosarcoma. However, PCAT6 also has different mechanisms in other tumors. For instance, PCAT6 endogenously competes with miRNAs to regulate gene expression and promote the progression of a variety of tumors. PCAT6 increases ZEB1 levels by sponging endogenous miR-143-3p, which supports the malignant phenotype of osteosarcoma cells. In breast cancer, PCAT6 acts as a sponge for miR-4723-5p to regulate VEGFR2, and also participates in the VEGFR/AKT/mTOR signaling pathway to accelerate angiogenesis. PCAT6 can function as a ceRNA to regulate the availability of miRNAs and regulate target gene expression in solid tumors.

Further studies have identified many proteins that interact with PCAT6. For example, HMGA2 is a binding partner of PCAT6 and contributes to PCAT6-mediated CRC, as well as EZH2 in CRC and lung cancer. These common or distinct molecular mechanisms and downstream signaling pathways promote malignant phenotypes, such as tumor proliferation, invasion, migration, and can stimulate the occurrence and development of tumors (**Table 1**). However, both the upstream and downstream regulatory mechanisms require further investigation to clarify the specific role of PCAT6 in tumors.

**Table 1:** Summary of the mechanism studies of lncRNA PCAT6 in cancers.

Tumor	Expression	Approach of function study	Downstream targets	Mechanism	Ref
Lung Cancer	up	Knockdown Overexpression	miR-330-5p	Regulating miR-330-5p	(11, 14–18)
			LATS2	Regulating LATS2	
			p53	Regulating p53	
			c-Myc	Regulating c-Myc	
Gastric Cancer	up	Knockdown Overexpression	MKRN3	Regulating miR-30/MKRN3 axis	(22, 23)
			RB/E2F	Targeting miR-15a to regulate RB/E2F and Wnt/β-catenin pathways	
Colorectal Cancer	up	Knockdown Overexpression	ARC	Regulating ARC expression *via* EZH2	(27, 28)
			HMGA2	Regulating miR‐204 to promote HMGA2/PI3K signaling activity	
Hepatocellular Carcinoma	up	Knockdown Overexpression	Not mentioned	Not mentioned	(31–34)
Bladder Cancer	up	Knockdown Overexpression	miR-513a-5p	Targeting miR-513a-5p	(38, 39)
Breast Cancer	up	Knockdown Overexpression	TPD52	Regulating miR-185-5p/TPD52 axis	(42, 43)
			VEGFR	Sponging miR-4723-5p to regulate VEGFR/AKT/mTOR signaling pathway	
Cervical Cancer	up	Knockdown Overexpression	ZEB1	Regulating miR-543/ZEB1 axis	(45, 46)
			β-catenin	Regulating Wnt/β-catenin signaling pathway	
Osteosarcoma	up	Knockdown Overexpression	MDM2	Regulating MDM2 expression	(54–57)
			TGFBR1/2	Regulating miR-185-5p-TGFBR1/2-TGF-β axis	
			ZEB1	Regulating miR-143-3p/ZEB1 axis	
GIST	up	Knockdown Overexpression	PRDX5	Sponging miR‐143‐3p to regulate PRDX5 and activating Wnt/β‐catenin pathway	(60)
Cholangiocarcinoma	up	Overexpression	miR-330-5p	Regulating miR-330-5p	(47)
Pancreatic Ductal Adenocarcinoma	up	Knockdown Overexpression	CBX2	Regulating miR-185-5p/CBX2 axis	(48)
Ovarian Cancer	up	Knockdown Overexpression	PTEN	Regulating PTEN	(49)
Glioblastoma	up	Knockdown Overexpression	IGF2BP1	Regulating miR-513/IGF2BP1 axis	(50)

For treatment and prognosis, abnormal expression of PCAT6 is related to poor clinicopathological features, as overexpression of PCAT6 often indicates poor prognosis (**Table 2**). It can likely affect the clinical efficacy of various cancer treatments. PCAT6 is a potential diagnostic biomarker, as well as a possible new prognostic biomarker. In past decades, cytotoxic chemotherapy has been developed, but has no significant advantages for overcoming drug resistance (62). However, accumulating studies have demonstrated that lncRNAs play important roles in chemotherapy resistance (63, 64). PCAT6 facilitated chemoresistance of CC cells to cisplatin *via* the PCAT6/miR-543/ZEB1 axis (46). PCAT6 also enhanced the chemoresistance of CRC cells to 5‐FU *via* the miR‐204/HMGA2/PI3K axis (28). PCAT6 also plays a critical role in radiotherapy. PCAT6 promoted the radiosensitivity of TNBC cells through regulating the miR-185-5p/TPD52 axis (43). According to these data, PCAT6 is expected to be a promising target for tumor therapy. These findings may provide new insight and a vital theoretical basis for the development of novel tumor treatments.

**Table 2:** LncRNA PCAT6 with tumor type, clinical significance and functions in cancers.

Tumor	Tumor type	Clinical significance	Function	Ref
Lung Cancer	carcinomas	Tumor size, TNM stage, Lymph node metastasis, Poor overall survival	Invasion, proliferation, apoptosis	(11, 14–18)
Gastric Cancer	carcinomas	Tumor size, TNM stage, Tumor node metastasis, Overall survival, EMT	Migration, invasion, apoptosis, proliferation	(22, 23)
Colorectal Cancer	carcinomas	Larger tumor size, Advanced TNM stages, Lymph node metastasis, Overall survival, 5‐fluorouracil‐based chemoresistance	Proliferation, apoptosis	(27, 28)
Hepatocellular Carcinoma	carcinomas	Advanced TNM stage, Poor overall survival, Poor disease-free survival, Cell cycle arrest	Proliferation, metastasis, apoptosis	(31–34)
Bladder Cancer	carcinomas	Tumor size, Differentiation, TNM stage, Lymph nodes metastasis, Shorter overall survival, Shorter progression-free survival	Proliferation, migration, invasion, apoptosis, distant metastasis	(38, 39)
Breast Cancer	carcinomas	Tumorigenesis, Angiogenesis, Tumor stage, Tumor growth, Lymph node metastasis, Cell survival, EMT process, Radiosensitivity	Apoptosis, proliferation, migration, invasion	(42, 43)
Cervical Cancer	carcinomas	Advanced FIGO stage, Positively lymph node metastasis, Overall survival, Disease-free survival, Cisplatin chemoresistance	Depth of invasion, proliferation, apoptosis	(45, 46)
Cholangiocarcinoma	carcinomas	Not mentioned	Proliferation, invasion	(47)
Pancreatic Ductal Adenocarcinoma	carcinomas	TNM stage, Lymph node invasion, Overall survival	Proliferation, migration, invasion	(48)
Ovarian Cancer	carcinomas	Lymph node metastasis	Proliferation, migration, invasion, distant metastasis	(49)
Glioblastoma	carcinomas	Survival	Proliferation, apoptosis	(50)
Osteosarcoma	sarcomas	Poor overall and progression-free survival	Metastasis, proliferation, migration, invasion	(54–57)
GIST	sarcomas	Not mentioned	Proliferation, apoptosis, stemness	(60)

Currently, several methods can be used to investigate the therapeutic targeting of upregulated lncRNAs in a tissue-specific manner, such as public databases, RNA sequencing, and qRT-PCR. These methods can help researchers further understand the downstream mechanisms, but also have some limitations. RNA sequencing is characterized as high throughput with moderate accuracy and sensitivity (65), while PCR is characterized as low throughput with high accuracy and sensitivity (66). When qRT-PCR is used, it is often unsuccessful if the tissue abundance of the RNA is low, causing signal amplification to be required. If fresh tissue samples had been used, the results could possibly be more reliable (67). All of these methods require invasive procedures to extract tissue. If serum analysis is used, it is not necessary to extract tissues repeatedly, which can be better applied in clinical practice. Additionally, the efficacy and prognosis can be dynamically detected, which has better clinical significance (68). However, the expression levels of PCAT6 in serum or malignant effusion in thoracic and abdominal cavity samples have not been clearly verified.

## Conclusions and Perspectives

The conclusions are based on the limited current research. Whether all tumors exhibiting similar high expression levels of PCAT6 are ideal targets for diagnosis and therapy remains largely unknown. In addition, the specific molecular mechanisms of PCAT6 in tumors are still not widely understood. Further research is needed to clarify this in more detail. Fortunately, following the accelerated advancement of biotechnology, the relationship between PCAT6 and various tumors can be identified and validated. We believe PCAT6 has the potential to eventually be applied in the clinical setting for oncology.

## Author Contributions

SW, ZC, and JG wrote and drafted the manuscript and figures. XC and ZW revised the manuscript. ZW and ZC designed this manuscript. All authors contributed to the article and approved the submitted version.

## Funding

This work was supported by grants from the National Natural Science Foundation of China (Nos., 82072591, 81871871 to ZW; 81902333 to XC), Key Research and Development plan (Social development) of science and technology department of Jiangsu Province (No. BE2019760), the Medical Innovation Team Foundation of the Jiangsu Provincial Enhancement Health Project (No. CXTDA2017021 to ZW), “123” advantageous disciplines, core technologies and “789” excellent talent training plan of the Second Affiliated Hospital of Nanjing Medical University (No. 789ZYRC202090146 to XC).

## Conflict of Interest

The authors declare that the research was conducted in the absence of any commercial or financial relationships that could be construed as a potential conflict of interest.
